# *NAA10* p.(D10G) and *NAA10* p.(L11R) Variants Hamper Formation of the NatA N-Terminal Acetyltransferase Complex

**DOI:** 10.3390/ijms21238973

**Published:** 2020-11-26

**Authors:** Nina McTiernan, Christine Darbakk, Rasmus Ree, Thomas Arnesen

**Affiliations:** 1 Department of Biomedicine, University of Bergen, N-5020 Bergen, Norway; Nina.Tiernan@uib.no (N.M.); rasmus.ree@uib.no (R.R.); 2 Department of Biological Sciences, University of Bergen, N-5020 Bergen, Norway; darbakk@hotmail.com; 3 Department of Surgery, Haukeland University Hospital, N-5021 Bergen, Norway

**Keywords:** N-terminal acetylation, NAA10, NAA15, NatA, acetyltransferase, genetic disease, syndrome, X-chromosome

## Abstract

The majority of the human proteome is subjected to N-terminal (Nt) acetylation catalysed by N-terminal acetyltransferases (NATs). The NatA complex is composed of two core subunits—the catalytic subunit NAA10 and the ribosomal anchor NAA15. Furthermore, NAA10 may also have catalytic and non-catalytic roles independent of NatA. Several inherited and de novo NAA10 variants have been associated with genetic disease in humans. In this study, we present a functional analysis of two de novo NAA10 variants, c.29A>G p.(D10G) and c.32T>G p.(L11R), previously identified in a male and a female, respectively. Both of these neighbouring amino acids are highly conserved in NAA10. Immunoprecipitation experiments revealed that both variants hamper complex formation with NAA15 and are thus likely to impair NatA-mediated Nt-acetylation in vivo. Despite their common impact on NatA formation, in vitro Nt-acetylation assays showed that the variants had opposing impacts on NAA10 catalytic activity. While *NAA10* c.29A>G p.(D10G) exhibits normal intrinsic NatA activity and reduced monomeric NAA10 NAT activity, *NAA10* c.32T>G p.(L11R) displays reduced NatA activity and normal NAA10 NAT activity. This study expands the scope of research into the functional consequences of NAA10 variants and underlines the importance of understanding the diverse cellular roles of NAA10 in disease mechanisms.

## 1. Introduction

N-terminal (Nt) acetylation is a pivotal protein modification occurring in a majority of eukaryotic proteins, either co- or post-translationally, which is catalysed by a family of eight N-terminal acetyltransferases (NATs) named NatA to NatH [1,2,3]. The chemical alteration introduced by Nt-acetylation can influence protein folding, complex formation, stability and degradation, and subcellular localisation [3,4,5,6,7,8,9,10,11].

NatA consists of the catalytic subunit NAA10 and the large auxiliary subunit NAA15 [12,13,14]. NAA15 changes the substrate specificity of NAA10 upon binding and anchors the complex to the ribosome [15,16,17,18]. HYPK is a stable interactor of the NatA complex and is thought to have an inhibitory role ensuring substrate specificity [19,20,21]. Among the NATs, NatA has the largest substrate pool and co-translationally Nt-acetylates alanine, serine, threonine, valine, cysteine and glycine after the initiator methionine has been cleaved off [1,18]. Monomeric NAA10 may also display NAT activity independent of NatA, with a distinct substrate specificity [15,18]. Furthermore, NAA10 has been described as a lysine acetyltransferase (KAT), acetylating lysine residues of substrates such as β-catenin, Hsp70 and SAMHD1 [22,23,24,25]. In addition to its catalytic roles, NAA10 may act as a non-catalytic regulator of STAT5a and DNMT1 [26,27,28].

The X-chromosomal *NAA10* gene is essential for normal cell function, and dysregulation of *NAA10* has been associated with several human cancers such as lung, breast, prostate and colon cancer [29,30]. Inherited or de novo missense variants in *NAA10* have also been identified as causative of genetic disease in humans [31]. Although individuals harbouring *NAA10* missense variants display phenotypic heterogeneity, the phenotypes observed in most affected individuals are developmental delay (DD), intellectual disability (ID) and cardiac anomalies. The severity of disease can range from lethal, as reported for males carrying *NAA10* c.109T>C p.(S37P), to only mild clinical manifestations in both males and females with different variants [32,33,34,35,36,37,38,39,40,41,42,43]. Functional studies have revealed that some *NAA10* variants, such as p.(Y43S), p.(I72T), p.(R83C), p.(R83H), p.(V107F), p.(V111G), p.(R116W), p.(F128L) and p.(F128I), negatively impact the catalytic NAT activity of monomeric NAA10 [35,36,37,38,41,42]. In contrast, *NAA10* p.(S37P), and recently p.(H16P) and p.(N101K), have been shown to impair NatA complex formation and NatA-mediated Nt-acetylation [33,39,43]. Moreover, three pathogenic *NAA10* variants, p.(S37P), p.(V107F) and p.(R116W), have been shown to disrupt NAA10 binding to imprinting control regions affecting DNMT1 recruitment and genomic imprinting [28]. Thus, different variants are likely to have pleiotropic effects depending on the functional impairment associated with each specific variant. The mechanisms underlying genetic diseases resulting from pathogenic *NAA10* variants remain elusive.

Here, we report the functional consequences of two de novo *NAA10* variants, c.29A>G p.(D10G) and c.32T>G p.(L11R). *NAA10* c.29A>G p.(D10G) was previously identified in a boy who died within 6 months of age [40]. The individual had DD, brain malformations, cardiac abnormalities, hearing loss and dysmorphic features. *NAA10* c.32T>G p.(L11R) was previously described in a young girl with DD, brain malformations and strabismus [40].

## 2. Results

### 2.1. Analysis of NAA10 Sequence and NatA Structure

**Figure 1:**
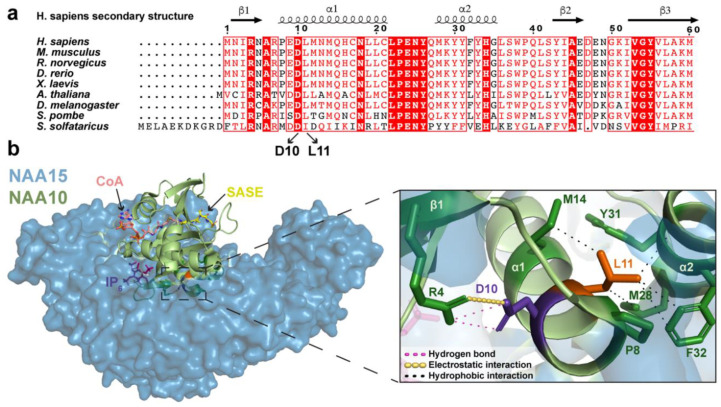
NAA10 multiple sequence alignment and human NatA structure. (**a**) A multiple sequence alignment of NAA10 sequences from *Homo sapiens*, *Mus musculus*, *Rattus norvegicus*, *Danio rerio*, *Xenopus laevis, Arabidopsis thaliana, Drosophila melanogaster*, *Schizosaccharomyces pombe* and *Saccharolobus solfataricus*. The amino acids D10 and L11 are highly conserved in the NAA10 sequences (conservation is coloured red). (**b**) NAA10 (green) and NAA15 (blue) in the human NatA structure (PDB ID: 6C9M) [21] bound with ligand IP_6_ (magenta). The human NatA structure was superimposed with the substrate (SASE, yellow sticks; CoA, pink sticks) from the *S. pombe* NatA structure (PDB ID: 4KVM) [15]. A zoomed-in view (right) shows D10 (purple) and L11 (orange) and the interactions they form with surrounding amino acids.

NAA10 is evolutionarily conserved from yeast to human [1]. A NAA10 multiple sequence alignment shows that the affected amino acids D10 and L11 are highly conserved, suggesting an important role in the function and/or stability of NAA10 (Figure 1a). The neighbouring residues D10 and L11 are located in the NAA10 α1 helix, which is part of the NAA10–NAA15 hydrophobic binding surface (Figure 1b). DynaMut [44] was used to identify potential interactions mediated by D10 and L11 with nearby amino acids. The negatively charged D10 was predicted to interact with the positively charged R4 in NAA10 β1 through electrostatic and hydrogen bonding. L11 was predicted to form a hydrophobic pocket together with P8, M14, M28, F32 and Y31. The residue R4 interacting with D10 and the hydrophobic residues interacting with L11 are also conserved in many NAA10 orthologues (Figure 1a), supporting a hypothesis that *NAA10* p.(D10G) and p.(L11R) variants may disrupt important interactions necessary for proper NAA10 function and/or stability. D10, L11 and the NAA10 residues they interact with were not predicted to directly interact with any NAA15 residues.

### 2.2. Complex Formation and Catalytic Activity

**Figure 2:**
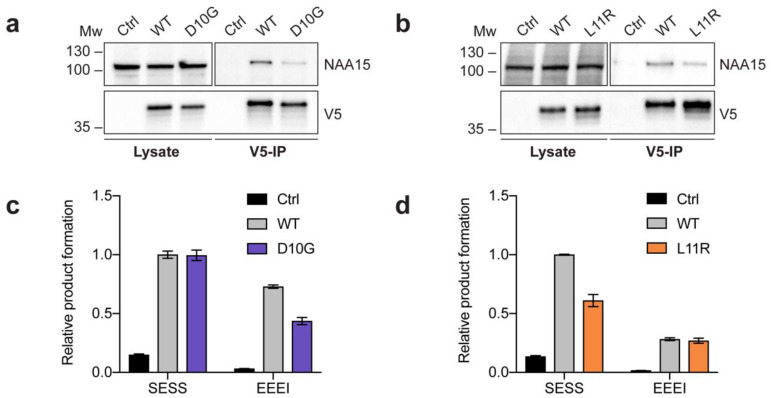
NAA10-V5 immunoprecipitation (IP) and in vitro N-terminal (Nt) acetylation assays. (**a**,**b**) Western blot analyses showing V5-IP of NAA10-WT-V5 and either NAA10-D10G-V5 (**a**) or NAA10-L11R-V5 (**b**). The immunoblots were probed with anti-V5 tag and anti-NAA15 antibodies. (**c**,**d**) The catalytic activity of the NAA10 variants, NAA10-D10G-V5 (**c**) and NAA10-L11R-V5 (**d**), was compared to NAA10-WT-V5 through in vitro Nt-acetylation assays. Negative controls included reactions without peptide and with β-gal-V5 IP. The measured activities towards the canonical NatA complex substrate SESS_24_ and the in vitro monomeric NAA10 substrate EEEI_24_ were normalised to the amount of NAA15 and NAA10-V5 used in the reaction, respectively. The Western blot analyses and Nt-acetylation assays shown are representative of three independent experiments. The Nt-acetylation assays were performed with three technical replicates.

To explore the impact of NAA10-D10G-V5 and NAA10-L11R-V5 on NatA complex formation and catalytic activity, HeLa cells overexpressing either the variants or wildtype NAA10 (NAA10-WT) were subjected to anti-V5 immunoprecipitation (IP), Western blot analysis and Nt-acetylation assays. A striking observation from the Western blot analyses was that less NAA15 co-immunoprecipitated with both variants as compared to NAA10-WT-V5 (Figure 2a,b; Figure S1). These results suggest that both variants impede NatA complex formation, which ultimately is likely to cause reduced NatA-mediated Nt-acetylation in vivo.

The catalytic activity of NAA10-D10G-V5 and NAA10-L11R-V5 was investigated through in vitro Nt-acetylation assays (Figure 2c,d). In these assays, SESS_24_ represented a canonical NatA substrate, while EEEI_24_ was used as a substrate for monomeric NAA10, which preferentially Nt-acetylates acidic N-termini in vitro. Therefore, the measured catalytic activity against SESS_24_ and EEEI_24_ was normalised to the amount of NAA15 and NAA10-V5, respectively. The Nt-acetylation assays revealed that NAA10-D10G-V5 displayed normal NatA catalytic activity, whereas the monomeric NAA10-D10G-V5 NAT activity was reduced by approximately two-fold as compared to NAA10-WT-V5 (Figure 2c). In contrast, NAA10-L11R-V5 demonstrated the opposite effect, the NatA catalytic activity being reduced compared to NAA10-WT-V5, while the monomeric NAT activity was not affected (Figure 2d).

In summary, the experimental evidence suggests that both variants hamper formation of the NatA complex but have different effects on the enzymatic activity of NAA10. NAA10-D10G-V5 showed normal activity in the formed NatA complex but impaired NAA10 monomeric activity, while NAA10-L11R-V5 showed impaired NatA activity but normal monomeric NAA10 activity.

## 3. Discussion

Functional characterisations of different NAA10 variants identified in individuals have revealed that distinct variants can affect the biological roles of NAA10 differently. We report here the functional characterisation of two de novo *NAA10* variants, p.(D10G) and p.(L11R). *NAA10* p.(D10G) and *NAA10* p.(L11R) were previously identified in a boy and a girl, respectively, with phenotypes including developmental delay and brain malformations [40].

Bioinformatic analyses of the NAA10 sequence and structure revealed that D10 and L11, as well as the amino acids they interact with, are highly conserved in NAA10 orthologues (Figure 1a,b), indicating that D10 and L11 are important for NAA10 stability or function. The missense variants *NAA10* p.(D10G) and *NAA10* p.(L11R) are likely to disrupt the electrostatic interaction between D10 and R4 and the hydrophobic pocket that L11 occupies, respectively. Thus, we hypothesised that these missense variants may cause structural changes that impede the NAA10 active site as well as the interactions between the NAA10 α1 helix and NAA15.

Biochemical experiments were performed to investigate how the variants impacted NatA complex formation and enzymatic activity. Immunoprecipitation of both variants revealed that they had reduced capacity to bind NAA15 compared to NAA10-WT-V5 (Figure 2a,b). This was probably caused by disruption of important interactions between the NAA10 α1 helix and NAA15 [15]. Furthermore, in vitro Nt-acetylation assays showed that NAA10-L11R-V5 had reduced NatA activity and NAA10-D10G-V5 had equal NatA activity compared to NAA10-WT-V5 (Figure 2c,d). Since both variants impair NatA complex formation, it is likely that they both result in less Nt-acetylation of NatA substrates at the ribosome in patient cells. Additionally, *NAA10* p.(L11R) probably showed a stronger decrease in Nt-acetylation of NatA substrates than *NAA10* p.(D10G) because of its debilitated NatA function, even in the formed NatA complex (Figure 2d).

The monomeric NAA10 NAT activity was only affected by the *NAA10* p.(D10G) variant and not *NAA10* p.(L11R). This suggests that *NAA10* p.(D10G) may affect the NatA-independent roles of NAA10, resulting in diverse downstream effects. To date, no in vivo NAT substrates for monomeric NAA10 have been identified. However, NAA10 has been reported to catalyse the lysine acetylation of several proteins as well as regulating proteins in a non-catalytic manner [3]. Whether these NAT-independent roles of NAA10 are affected by the *NAA10* p.(D10G) or *NAA10* p.(L11R) variants remains unknown.

The *NAA10* p.(D10G) and *NAA10* p.(L11R) variants were previously reported by Cheng et al. in an international cohort of individuals with NAA10 variants [40]. In this cohort study, both variants were found to cause a decreased in vitro thermostability of NatA compared to NatA WT, which is in accordance with our IP evidence showing that both variants have a decreased binding affinity for NAA15 in vivo. Furthermore, Cheng et al. tested the in vitro hNatA catalytic activity of NAA10 variants purified from insect Sf9 cells. While both *NAA10* p.(D10G) and *NAA10* p.(L11R) were found to have reduced NatA activity, *NAA10* p.(D10G) was more strongly affected than *NAA10* p.(L11R). This contradicts our findings in which *NAA10* p.(D10G) NatA activity was unaffected in the formed NatA complex. One explanation for this discrepancy could lie in the nature of the experiment; we tested NatA immunoprecipitated from human cells in which potential binding partners and/or post-translational modifications not present in insect cells could influence enzymatic activity. HYPK is a known binding partner of NatA. In fact, Cheng et al. found that the NatA activity of *NAA10* p.(L11R) was not reduced when bound to HYPK, which highlights the intricacies of determining the impact of *NAA10* variants on the NatA machinery.

Since males only have one X-chromosome, they are generally more severely affected by *NAA10* variants than heterozygous females. The boy harbouring *NAA10* p.(D10G) died during infancy and had similar clinical features to males harbouring *NAA10* p.(S37P)—a lethal condition originally called Ogden syndrome [32]. Similar to *NAA10* p.(D10G) and p.(L11R), *NAA10* p.(S37P) has been found to impair NatA complex formation and cause reduced Nt-acetylation of some NatA substrates in Ogden-syndrome patient fibroblasts and B-cells. Female carriers of *NAA10* p.(S37P) had no severe symptoms, which is possibly explained by their X-chromosome inactivation pattern favouring the WT allele [33]. The impact of X-chromosome inactivation skewing on the clinical manifestations in the female carrying *NAA10* p.(L11R) is unclear, as it has not been experimentally tested. Recently, a de novo *NAA10* p.(H16P) variant was identified in a young female with severe ID and DD [39]. The *NAA10* p.(H16P) variant was located in the NAA10 α1 helix and functional studies revealed that this variant also hampered NatA complex formation. Furthermore, the *NAA10* p.(H16P) enzymatic activity resembled that of *NAA10* p.(L11R), in that the NatA activity was reduced but the monomeric NAA10 NAT activity was intact. Another *NAA10* variant, p.(N101K), was recently described in two unrelated girls with DD and hemihypertrophy [43]. The *NAA10* p.(N101K) variant was shown to completely abolish NatA complex formation and NatA enzymatic activity, while its monomeric NAA10 NAT activity was unaffected. *NAA10* p.(L11R) may thus have a similar disease mechanism as *NAA10* p.(H16P) and *NAA10* p.(N101K), in which the phenotypes are caused by impaired NatA Nt-acetylation at the ribosomes.

In conclusion, *NAA10* p.(D10G) and *NAA10* p.(L11R) appear to be causative of disease through decreased NatA complex formation and consequently reduced NatA-mediated Nt-acetylation of NatA substrates. Since NatA has a large substrate pool, approximately 40% of the human proteome [3], it is challenging to define all the downstream effects this can cause. *NAA10* p.(D10G) may additionally impede the individual roles of NAA10 in the cell. More research is needed in order to unravel the exact disease mechanisms involved with pathogenic NAA10 variants and understand their effect on NAT, KAT and the non-catalytic functions of NAA10.

## 4. Materials and Methods

### 4.1. Construction of Vectors

Vectors containing the NM_003491.3 *NAA10* c.29A>G p.(D10G) and *NAA10* c.32T>G p.(L11R) variants were generated by mutagenesis of the mammalian expression vector pcDNA3.1/*NAA10*-V5 using the Q5 Site-Directed Mutagenesis Kit (New England Biolabs, Ipswich, Massachusetts, US) protocol. The primers used for *NAA10* c.29A>G p.(D10G) were 5′-AGGCCAGAGGGCCTAATGAAC-3′ and 5′-CGCATTGCGGATGTTCATC-3′. The primers used for *NAA10* c.32T>G p.(L11R) were 5′-CCAGAGGACCGAATGAACATGC-3′ and 5′-CCTCGCATTGCGGATGTT-3′. The variants were verified by sequencing.

### 4.2. Bioinformatic Analyses

A NAA10 multiple sequence alignment was created using the alignment tool Clustal Omega [45] and annotated with sequence conservation and secondary structure information using ESPript 3.0 (http://espript.ibcp.fr) [46]. The NAA10 sequences used were from *Homo sapiens* (Uniprot ID: P41227), *Mus musculus* (Uniprot ID: Q9QY36), *Rattus norvegicus* (Uniprot ID: D3ZUQ2), *Danio rerio* (Uniprot ID: Q7T3B8), *Xenopus laevis* (Uniprot ID: Q7ZXW3), *Arabidopsis thaliana* (Uniprot ID: Q9FKI4), *Drosophila melanogaster* (Uniprot ID: Q9VT75), *Schizosaccharomyces pombe* (Uniprot ID: Q9UTI3) and *Saccharolobus solfataricus* (Uniprot ID: Q980R9). PyMOL (version 2.3.1, Schrödinger Inc., New York, NY, US) was used to visualise the human NatA structure (PDB ID: 6C9M) [21] and to superimpose it with the substrate SASE and CoA from *S. pombe* NatA (PDB ID: 4KVM) [15]. DynaMut [44] was used to predict molecular interactions.

### 4.3. Transfection, Immunoprecipitation and Western Blot Analysis

Five 10 cm dishes of HeLa cells (ATCC CCL-2) were transiently transfected with either 4 µg of WT pcDNA3.1/*NAA10*-V5 and 2–4 µg of empty pcDNA3.1/V5 vector, 8 µg of pcDNA3.1/*NAA10*-D10G-V5, 6 µg of pcDNA3.1/*NAA10*-L11R-V5 or 6–8 µg of pcDNA3.1/*LacZ*-V5 vector (negative control). Transfection was performed according to the X-tremeGENE 9 DNA Transfection Reagent (Roche, Basel, Switzerland) protocol. After 24 h, the cell medium was replaced and the cells were harvested after 48 h. The harvested cells were lysed in 1 mL IPH lysis buffer (50 mM Tris-HCl pH 8.0, 150 mM NaCl, 5 mM EDTA, 0.5% NP-40, 1× complete EDTA-free protease inhibitor cocktail (Roche)) for 15 min at 4 °C on a rotating wheel. Cell debris was removed by centrifugation (17,000× *g*, 4 °C, 5 min) and the resulting supernatant was incubated with 4 µg of V5-tag mouse monoclonal antibody (Invitrogen, R960-25, Carlsbad, CA, US) at 4 °C for 2 h on a rotating wheel. Subsequently, 40 µL of pre-washed Dynabeads Protein G (Invitrogen) was added to each sample and incubated overnight. The beads were washed three times in IPH lysis buffer, re-suspended in 90 µL acetylation buffer (50 mM Tris-HCl pH 8.5, 1 mM EDTA, 10% Glycerol) and used in the Western blot analyses and Nt-acetylation assays. Immunoblots were probed with V5-tag mouse monoclonal antibody (1:5000 dilution, Invitrogen, R960-25) and NAA15 rabbit polyclonal antibody (1:2000 dilution, BioGenes (Berlin, Germany) [12]). The resulting protein bands were imaged and quantified using ChemiDoc XRS+ System (Bio-Rad, Hercules, CA, US) and Image Lab Software (version 6.0.1, Bio-Rad, Hercules, CA, US).

### 4.4. In Vitro N-terminal (Nt)-Acetylation Assays

In vitro Nt-acetylation assays were performed as described [47]. Triplicate reactions were run in each assay, and contained the following: 10 µL immunoprecipitated enzyme, 200 µM synthetic oligopeptide SESS_24_ (SESSSKSRWGRPVGRRRRPVRVYP) or EEEI_24_ (EEEIAALRWGRPVGRRRRPVRVYP) (>95% purity, BioGenes), 50 µM [14C]-Ac-CoA (PerkinElmer, Waltham, MA, US) and acetylation buffer to a final volume of 25 µL. Negative control reactions contained either immunoprecipitated β-gal-V5 or no substrate peptide. Reactions were incubated at 37 °C for 30 min on a thermoshaker (Eppendorf, Hamburg, Germany). To measure product formation, the reaction was quenched by transferring 23 µL of the reaction mixture onto P81 phosphocellulose filter discs (Millipore, Burlington, MA, US). The filter discs were washed three times in 10 mM HEPES buffer (pH 7.4) and dried before adding them to 5 mL Ultima Gold F scintillation mixture (PerkinElmer). Product formation was measured by a TriCarb 2900TR Liquid Scintillation Analyzer (PerkinElmer). The two variants were tested in independent setups compared to the WT enzyme, thus explaining some variability in the relative activity of WT NAA10 towards different substrates.

## Supplementary Materials

Supplementary materials can be found at https://www.mdpi.com/1422-0067/21/23/8973/s1.

## Abbreviations

Nt	N-terminal
NAT	N-terminal acetyltransferase
KAT	lysine acetyltransferase
DD	developmental delay
ID	intellectual disability
IP	immunoprecipitation
