# Supplementary figures and images for: NAA10 p.(D10G) and NAA10 p.(L11R) Variants Hamper Formation of the NatA N-Terminal Acetyltransferase Complex
Source: Int J Mol Sci. 2020 Nov 26;21(23):8973. doi: 10.3390/ijms21238973 (PMC7730585; doi:10.3390/ijms21238973)

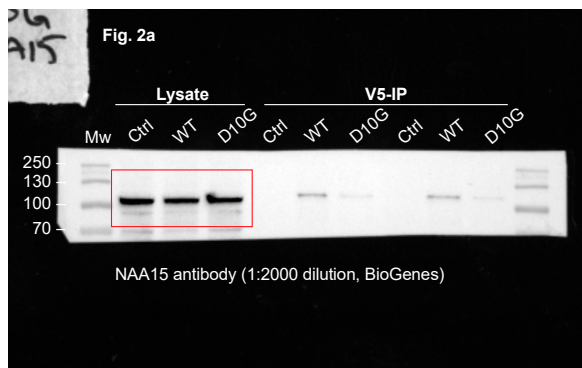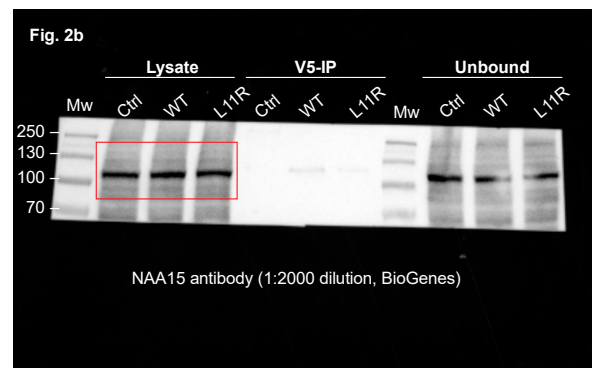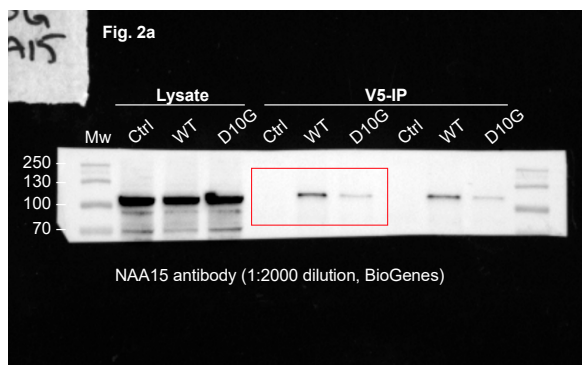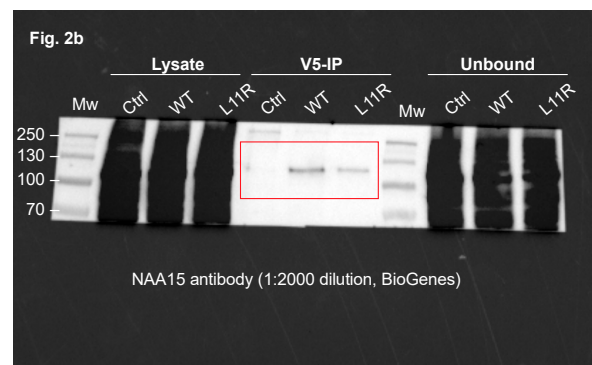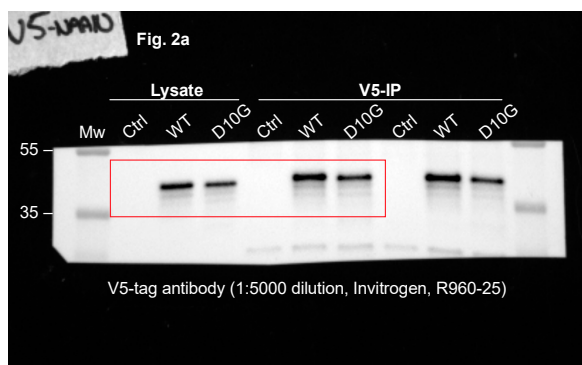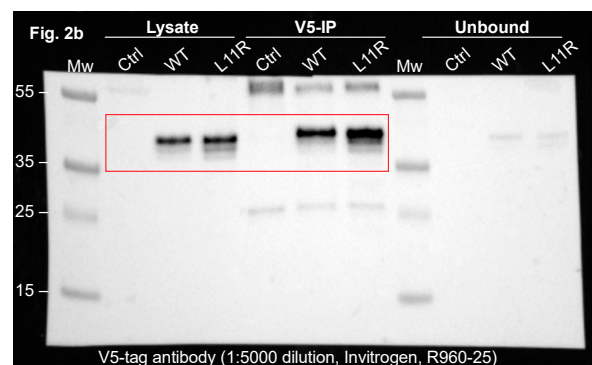

**Figure S1.** Original Western blot images used in main Figure 2.

Supplement: Supplementary file 1 [file ijms-21-08973-s001.pdf]
